# p33ING1b regulates acetylation of p53 in oral squamous cell carcinoma via SIR2

**DOI:** 10.1186/s12935-020-01489-0

**Published:** 2020-08-18

**Authors:** Xiao-han Li, Dan Li, Chang Liu, Ming-ming Zhang, Xiao-jiao Guan, Ya-ping Fu

**Affiliations:** Department of Pathology, Shengjing Hospital of China Medical University, No 36 Sanhao Street, Shenyang, 110004 Liaoning China

**Keywords:** OSCC, p33ING1b, p53, SIR2, SIRT1

## Abstract

**Background:**

Oral squamous cell carcinoma (OSCC), a form of head and neck squamous cell carcinoma (HNSCC) has a poor 5-year survival rate. OSCC patients are often treated with cisplatin but resistance to chemotherapy is often observed. This makes it important identification of alternative therapeutic targets which will result in more favorable outcome in OSCC patients. The plant homeodomain (PHD)-containing protein Inhibitor of Growth family of tumor suppressor proteins (p33ING1b) has been indicated as a tumor suppressor in different cancers including OSCC. This protein has been shown to function by modulating transcriptional activity of p53; however, the exact mechanism(s) are not well defined.

**Methods:**

Expression of total and acetylated p53 and p33ING1b protein was determined in OSCC cell lines YD-9, YD-8, and YD-38 by immunoblot analysis. Effect of modulation of p33ING1b protein expression on acetylation of p53 and cell proliferation was determined by immunoblot and MTT assay. Effect of modulation of p33ING1b protein expression on transactivation of p53 was assessed by heterologous promoter-based reporter and chromatin immunoprecipitation. Effect of modulation of expression of p33ING1b on SIR2 mRNA and protein was determined by quantitative real-time PCR and immunoblot analyses. Impact of modulation of p33ING1b alone or in combination with SIR2 on chemosensitivity of YD-9 and YD-8 cells to cisplatin was determined in time and dose-dependent cell proliferation assays.

**Results:**

Here, using a panel of OSCC cell lines with wild type or mutant p53, we show that p33ING1b expression is correlated to acetylation of p53 at lysine 382 residue. Increased acetylation of p53 following overexpression of p33ING1b was associated with increased expression of the pro-apoptotic proteins BAX, p21, and cleaved-Caspase 3, and decreased cell proliferation. Reporter assays with p21 and BAX promoters showed that p33ING1b expression levels directly correlated to promoter activity of these 2 genes. Chromatin immunoprecipitation assay showed that transcriptional regulation of p21 and BAX by acetylated p53 is dependent on expression level of p33ING1b. Differential acetylation of p53 following modulation of p33ING1b expression was indirect. Expression of p33ING1b was found to be inversely correlated to the NAD-dependent deacetylase silent information regulator 2 (SIR2). SIR2 was transcriptionally regulated by p33ING1b. Relative expression of p33ING1b was found to dictate chemosensitivity of OSCC cell lines to cisplatin treatment. Concomitant overexpression of p33ING1b and knockdown of SIR2 had a synergistic effect on chemosensitivity of OSCC cell lines to cisplatin, compared to either overexpression of p33ING1b or knockdown of SIR2 alone.

**Conclusions:**

The results from the current study thus elucidate that p33ING1b regulates p53 acetylation irrespective of p53 mutation and subsequent transactivation by transcriptional regulation of SIR2 expression. The results also indicate that p33ING1b and SIR2 are potentially attractive therapeutic targets.

## Background

One of the important groups of plant homeodomain (PHD)-containing proteins are the Inhibitor of Growth family of tumor suppressor proteins (ING1-5) [1]. The ING proteins are evolutionarily conserved and have been indicated to play functional role in a host of regulatory pathways inclusive of cell cycle, apoptosis, senescence, chromatin remodeling, and DNA damage response [1]. The major isoform of ING1 in normal as well as transformed cells is p33ING1b [2].

ING1b is an essential component of the nuclear Sin3-HDAC complex where it interacts via the PHD domain with H3K4Me3 [3–5]. Interaction of ING1b with H3K4Me3 and HDAC at the same time initiates DNA damage response signaling by causing local histone deacetylation and transcriptional inhibition. Alternatively, it interacts with proliferating nuclear cell antigen (PCNA) and mediates pro-apoptotic signaling following genotoxic stress [6, 7]. Indeed, p33ING1b overexpression has been shown to result in pro-apoptotic signaling [2, 8, 9].

Given its tumor suppressor role, inactivation of nuclear function of p33ING1b is often found in cancers, even though genetic inactivation is rarely reported [10–15]. Inactivation of p33ING1b function has largely been attributed to the tyrosine kinase Src and 14-3-3 proteins [16, 17]. Nuclear to cytoplasmic shuttling and interaction with mitochondria has also been indicated to play a role in p33ING1b-mediated pro-apoptotic signaling [1]. A major part of p33ING1b function role is mediated by activation of p53 signaling [18], where the former can stabilize p53 by preventing its interaction with the E3 ligase MDM2 [19].

Oral squamous cell carcinoma (OSCC) is a type of head and neck squamous cell carcinoma (HNSCC) with more than 250,000 new cases each year globally [20, 21]. The 5-year survival is a low 50% [20, 21]. Cisplatin is routinely used to treat OSCC patients, but resistance to cisplatin treatment is often observed in these patients [22]. Both decreased expression and altered cytoplasmic localization of p33ING1b has been correlated to poor prognosis in OSCC [23–26]. Another member of ING family, ING2, has been shown to modulate p53 function by acetylation [27].

The objective of our work was to determine if p33ING1b regulates acetylation and transactivation of p53 signaling in OSCC cell lines, harboring either wild type or mutant p53. Our results indicate that ING1b regulates p53 acetylation and subsequent activation of pro-apoptotic signaling in OSCC cell lines, irrespective of their p53 mutation status. Effect of p33ING1b on p53 acetylation was found to be mediated by the NAD-dependent deacetylase silent information regulator 2 (SIR2). Importantly, concomitant modulation of p33ING1b and SIR2 had a synergistic effect on in vitro chemosensitivity to cisplatin.

## Methods

### Cell culture

The OSCC cell lines YD-9 (60,502, buccal mucosa – wild type p53), YD-8 (60,501, tongue—point mutation at codon 273 of exon 8 of p53 (p.R273H)), and YD-38 (60,508, lower gingiva – p53 null) were purchased from Korean Cell Line Bank (Seoul, Korea) [28]. The OSCC cell lines Ca9-22 (p.R248W mutation in p53) and Sa-3 (p.R248Q mutation in p53). Were purchased from RIKEN BioResource Center (Ibaragi, Japan). All three cell lines were maintained in RPMI1640 medium containing 10% FBS (Thermo Fisher Scientific). Cells were maintained at 37 °C in incubator containing 5% carbon dioxide.

### Plasmids, transduction, transfection and luciferase assay

Expression plasmid for ING1b (pCI-ING1b) and SIR2 (FLAG-SIRT1) was obtained from Addgene (#79052 and #1791, respectively). Cells were stably transduced with MISSION pLKO.1-puro Non-Mammalian shRNA Control Plasmid DNA (#SHC002; Sigma-Aldrich, Shanghai, China) or MISSION *ING1b* shRNA Lentiviral Transduction Particles (#SHCLNV-NM_005537; Sigma-Aldrich) using polybrene and selected using puromycin (2 µg/ml) for 2 weeks. To generate SIR2 shRNA 5′-GGGAATCCAAAGGATAATT-3′ and 5′-AATTATCCTTTGGATTCCC-3′ were synthesized, annealed, and cloned into lentivirus vector GV248. YD-9 cells and YD-9 cells stably expressing ING1b shRNA were transduced with SIR2 shRNA as described above and selected using G418 (500 µg/ml) for 3 weeks. *BAX* luciferase reporter construct was generated by subcloning the PCR-generated fragment (− 715 bp to − 317 bp of *BAX* promoter) from genomic DNA into BglII and HindIII sites of the pGL3-luciferase Enhancer vector (Promega, Shanghai, China). The *CDKN1A* luciferase reporter (pGL2-p21 promoter-Luc) was obtained from Addgene (#33,021, Cambridge, USA). pRL-SV40, expressing renilla luciferase, was purchased from Promega and used as a transfection control for all luciferase reporter assays. Polyplus jetPrime transfection reagent was used to transfect 4 × 10^4^ cells with 0.5 µg each of firefly reporter plasmid and control renilla plasmid. Twenty-four hours post-transfection luciferase assay was performed using the Dual Luciferase Assay kit (Promega). Firefly luciferase values for each well were divided by corresponding renilla luciferase values and relative reporter activity (relative luminescence units, RLU) was plotted.

### Cell proliferation assay

Cell proliferation was determined using the MTT assay kit (Sigma Millipore, USA). Absorbance was measured at 570 nm. Cell proliferation was calculated as = (day 2, 3, or 4 mean – day 0 mean)/day 0 mean. For calculating cell viability post-Cisplatin treatment, percent cell viability was calculated as = (cisplatin group mean – DMSO group mean)/DMSO group mean * 100%.

### Western blot

Cell lysates were prepared in RIPA buffer (Thermo Fisher Scientific). Lysates were run on SDS-PAGE gels. Antibodies (all antibodies were used at 1:1000 dilution) used were p53, Acetyl-p53(K382), ING1b, Bax, SIR2 (SIRT1), p21, Cleaved Caspase-3, Bcl-xL, and GAPDH (Cell Signaling, Cambridge, MA, USA).

### Chromatin immunoprecipitation (ChIP)

Nuclear proteins were crosslinked to genomic DNA in about 3 million cells using in 1% (v/v) formaldehyde for 10 min at room temperature. Cells were lysed in SDS Lysis Buffer (Upstate, #20–163). Post-sonication, samples were centrifuged at 15,000*g*. Post-centrifugation the supernatants were diluted in ChIP Dilution Buffer (Upstate, #20–153). Immunoprecipitation was performed overnight at 4 °C with either 2 µg of anti- Acetyl-p53(K382) or normal anti-IgG antibody (control). Post-incubation, beads were washed (5 min each wash) in low-salt, high-salt, and LiCl buffers (Upstate, #20–154, #20–155, and #20–156, respectively). All washes were done at 4 °C. The beads were then washed twice (2 min at room temperature) in 1x TE (Upstate, #20–157). 1% SDS/100 mM NaHCO_3_ was used to elute the DNA from the beads. The immunoprecipitated DNA and serial dilutions of the input DNA were analyzed using real-time PCR using the following primers specific for the respective promoter loci: *CDKN1A* Forward: 5′-GCTCATTCTAACAGTGCTGTG-3′; *CDKN1A* Reverse: 5′-CAAGGAACTGACTTCGGCAG-3′; *BAX* Forward: 5′-GCCTGGGCAACACAGTGAG-3′; and, *BAX* Reverse: 5′-GCTCCCTCGGGAGGTTTG-3′ and the following primers specific for regions downstream of the respective promoter loci as a negative control to rule out false positive resulting from inefficient DNA fragmentation: *CDKN1A* Forward: 5′-GCCTTGCAGGAAACTGACTC-3′; *CDKN1A* Reverse: 5′-GGCTCTCATAGGCCTCTCCT-3′; *BAX* Forward: 5′- GCGATCTCCAAGCACTGAG-3′; *BAX* Reverse: 5′- GGGATCAGAGAGCCAGGAAC-3′.

### Isolation of total RNA and quantitative real time PCR

Total RNA from YD-9 cells was isolated using PureLink RNA Mini kit (Thermo Fisher) and treated with DNase (Thermo Fisher). First strand cDNA was synthesized using SuperScript III (Thermo Fisher). Second strand PCR was done using the PowerTrack SYBR Green Master mix (Thermo Fisher) and primers specific for *SIR 2* (Forward: 5′- TAGACACGCTGGAACAGGTTGC-3′ and Reverse: 5′-CTCCTCGTACAGCTTCACAGTC-3′) and *GAPDH* (Forward: 5′-GTCTCCTCTGACTTCAACAGCG-3′; Reverse: 5′-ACCACCCTGTTGCTGTAGCCAA-3′). *SIR2* expression was normalized to *GAODH* expression and relative expression was calculated using the 2^− ΔΔCt^ method. Data was presented as mean ± standard deviation (SD)) of three biological replicates, each done in three technical replicates.

### Statistical analysis

All data was represented as mean ± SD of at least three independent replicates. Statistical significance between groups were analyzed using the Student’s t-test. A p-value < 0.05 was considered statistically significant.

## Results

**Fig. 1:**
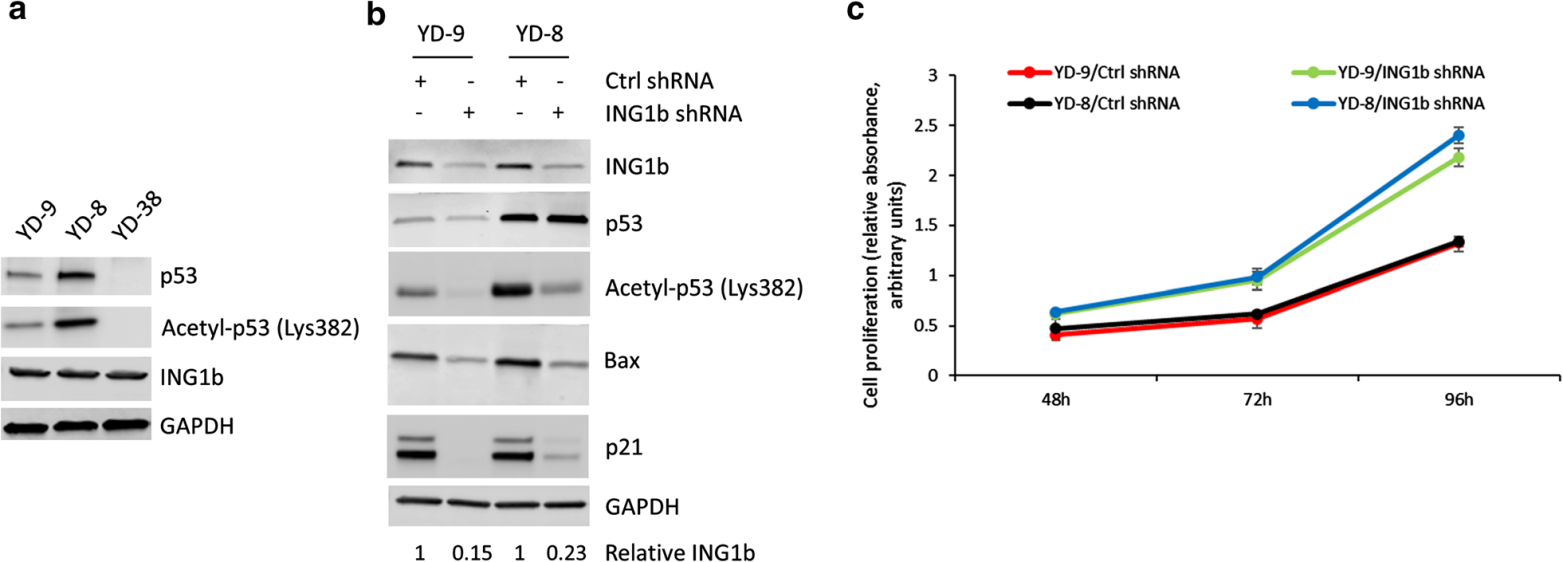
ING1b regulates p53-acetylation and downstream activation of pro-apoptotic pathway. **a** Relative protein expression of p53, acetylated p53 (at Lys382) and ING1b in OSCC cell lines YD-9 (wild type *TP53*), YD-8 (*TP53* point mutation), and YD-38 (*TP53* deletion). GAPDH was used as a loading control. Data is representative of three independent experiments. **b** Immunoblot analysis of total and acetylated p53 (at Lys382), and pro-apoptotic Bax and p21 in YD-9 and YD-8 cells stably transduced with control or *INGb1* shRNA shows ING1b regulates acetylation and downstream expression of pro-apoptotic proteins. GAPDH was used as a loading control. Data is representative of three independent experiments. Numbers below the figure shows relative expression of ING1b as determined by densitometry analysis. **c** Cell proliferation was assayed for 3 days in YD-9 and YD-8 cells transiently transfected with control or *ING1b* shRNA, 48 h after transfection. Data represented in from 3 different independent experiments. Error bars, SD

In order to determine if there is a correlation between p53 mutation status, acetylation of p53 at lysine 382, and basal expression of p33ING1b, we initially determined protein expression of p33ING1b and p53 in the OSCC cell lines YD-9 (buccal mucosa—wild type p53), YD-8 [tongue—point mutation at codon 273 of exon 8 of p53 (R273H)], and YD-38 (lower gingiva—p53 null). There was no difference in basal expression of p33ING1b between the three cell lines (Fig. 1a). Relative expression of p53 and acetylated p53 was higher in the YD-8 cells. As expected, no p53 expression was detected in the YD-38 cells (Fig. 1a).

We next determined if the acetylation of p53 and transactivation of prop-apoptotic proteins Bax and p21 in the YD-9 and YD-8 cells was dependent on p33ING1b expression. YD-9 and YD-8 cells were stably transduced with either control or *ING1b* shRNA. Successful knockdown of ING1b was verified by western blot (Fig. 1b, *top panel*). Knockdown of p33ING1b downregulated acetylation of p53 as well as expression of Bax and p21 in both YD-9 and YD-8 cell lines (Fig. 1b). Knockdown of p33ING1b had no effect on total p53 expression. Knockdown of p33ING1b significantly increased cell proliferation in both YD-9 and YD-8 cell lines (Fig. 1c). These results indicated that ING1b protein expression level is correlated to acetylation and transactivation of p53 in the context of the tested OSCC cell lines, irrespective of whether it is wild-type or mutant p53.

**Fig. 2:**
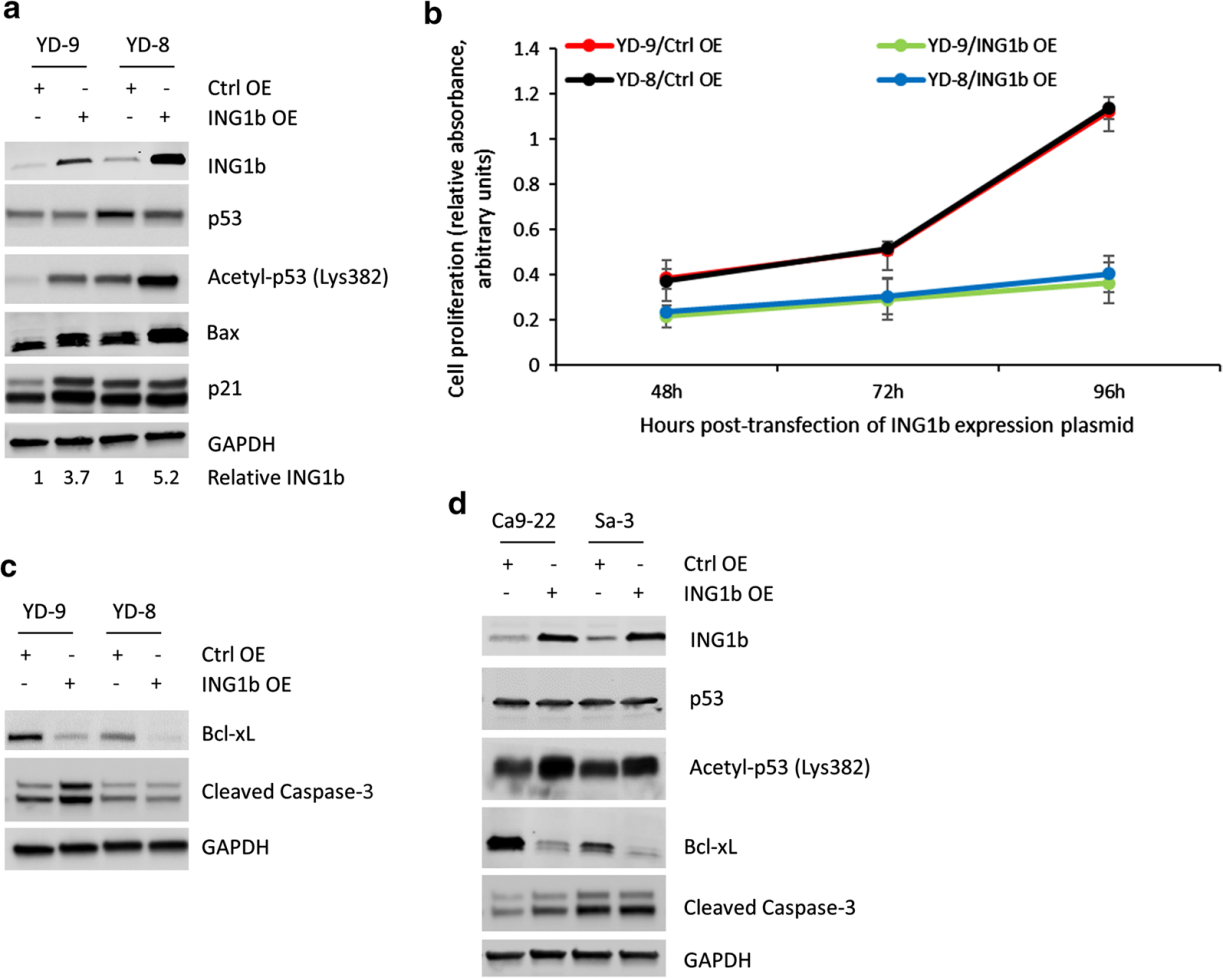
Over expression of ING1b increase acetylated p53, decrease cell proliferation, and induce apoptosis. **a** Immunoblot analysis of ING1b, total and acetylated p53 (at Lys382), and pro-apoptotic Bax and p21 in YD-9 and YD-8 cells transiently transfected with control or *ING1* expression plasmid. GAPDH was used as a loading control. Data is representative of three independent experiments. Numbers below the figure shows relative expression of ING1b as determined by densitometry analysis. **b** Cell proliferation was assayed for 3 days in YD-9 and YD-8 cells transiently transfected with control or *ING1* expression plasmid, 48 h after transfection. Data represented in from 3 different independent experiments. Error bars, SD. **c** Immunoblot analysis of anti-apoptotic Bcl-XL and pro-apoptotic Cleaved Caspase-3 in YD-8 and YD-9 cells transiently transfected with control or ING1b expression plasmids. GAPDH was used as a loading control. Data is representative of three independent experiments. **d** Immunoblot analysis of ING1b, total and acetylated p53 (at Lys382), anti-apoptotic Bcl-XL and pro-apoptotic Cleaved Caspase-3 in Ca9-22 and Sa-3 cell lines transiently transfected with control or ING1b expression plasmids. GAPDH was used as a loading control. Data is representative of three independent experiments

In order to confirm the role of ING1b in acetylation of p53 and activation of pro-apoptotic pathway we next overexpressed ING1b in the YD-9 and YD-8 cells (Fig. 2a, *top panel*). Overexpression of ING1b resulted in increase of acetylated p53, but not total p53 protein (Fig. 2a). This increase in p53 acetylation was accompanied by increased expression of Bax and p21 expression (Fig. 2b), as well as significant decrease in cell proliferation over 3 days (Fig. 2b; P < 0.05 in each case). Overexpression of p33ING1b also resulted in downregulation of the anti-apoptotic protein Bcl-xl in both YD-9 and YD-8 cells and increased expression of cleaved Caspase-3 in YD-9 cells (Fig. 2c), indicating that p33ING1b overexpression increased p53 acetylation and subsequent suppression of pro-proliferative pathways. Of note, the effect was more promiscuous in the YD-9 cells with wild type p53 compared to YD-8 cells with the p53 point mutation, perhaps partially due to saturated expression of Bax and p21 in the YD-8 cells in the basal condition. No apoptosis difference for ING1b overexpression was observed in YD-8 cells in respect to cleaved Caspase-3, which might suggest the mechanism is different between YD-9 and YD-8 cells. To determine if that is the case, we overexpressed ING1b in two additional OSCC cell lines, Ca9-22 (p.R248W mutation in p53) and Sa-3 (p.R248Q mutation in p53) (Fig. 2d, *top panel*). ING1b overexpression resulted in increase of acetylated p53, but not total p53 protein (Fig. 2d). This increase in p53 acetylation was accompanied by robust decrease in expression of the anti-apoptotic protein Bcl-xl (Fig. 2d). Like YD-8 cells, the increase in cleaved Caspase-3 was modest in Ca9-22 and Sa-3 cells. Taken together, these results confirmed that ING1b overexpression results in elevated acetylation of p53 and subsequent inhibition of cell proliferation.

**Fig. 3:**
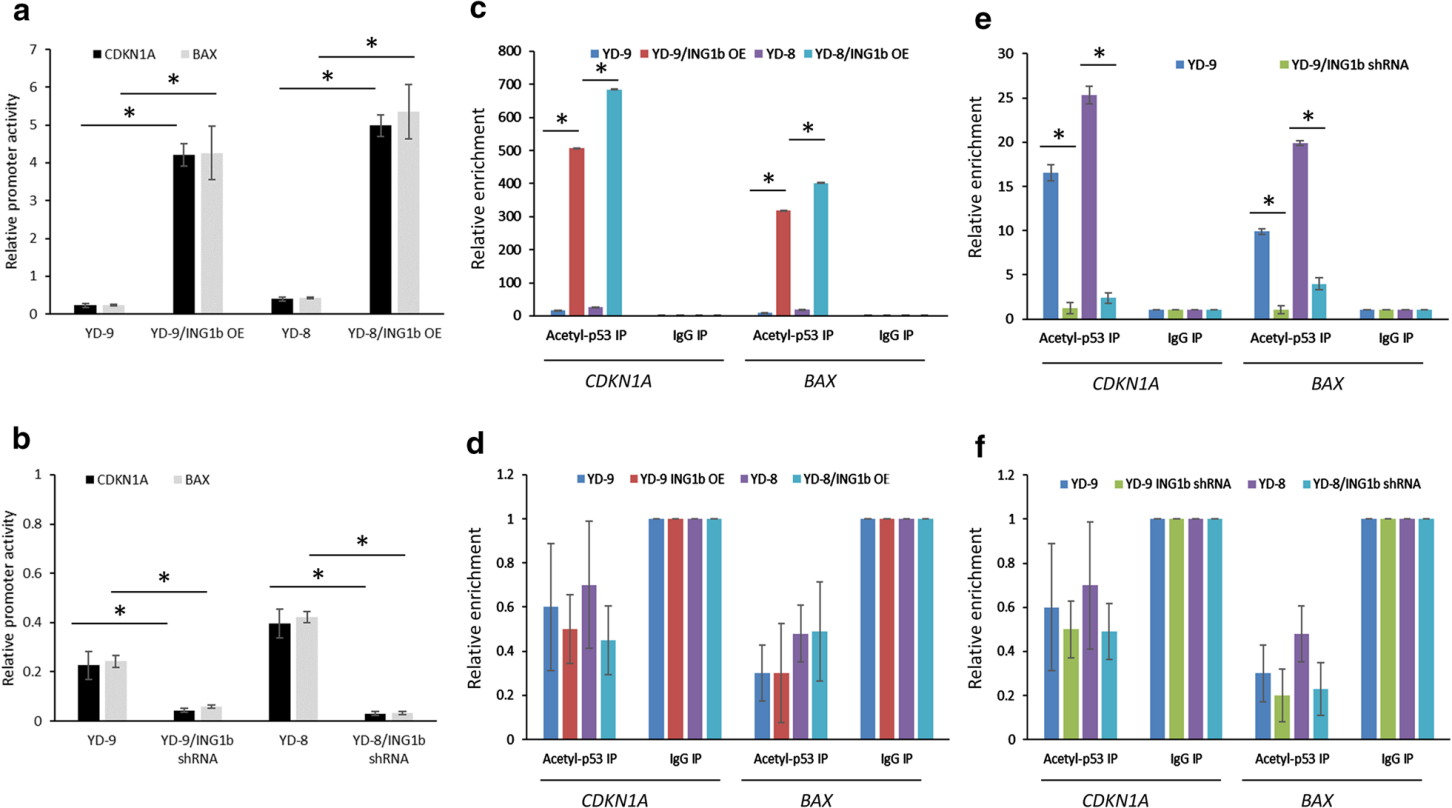
ING1b expression is correlated to p53-mediated p21 and Bax transcriptional activity. **a** Firefly luciferase promoter reporters for *CDKN1A* (encoding p21) or *BAX* and Renilla luciferase control reporters were co-transfected in YD-9 and YD-8 cells 48 h post-transfection with either control or ING1b expression plasmid. Dual luciferase assay was performed 24 h post-transfection of luciferase reporters. Data was normalized to Renilla luciferase. Error bars, SD. *P < 0.05. **b** Firefly luciferase promoter reporters for *CDKN1A* (encoding p21) or *BAX* and Renilla luciferase control reporters were co-transfected in YD-9 and YD-8 cells stably transduced with control or *ING1b* shRNA. Dual luciferase assay was performed 24 h post-transfection of luciferase reporters. Data was normalized to Renilla luciferase. Error bars, SD. *p < 0.05. **c** Chromatin immunoprecipitation assay (ChIP) shows direct binding of acetylated-p53 to both *CDKN1A* and *BAX* promoters, which was significantly more following transient overexpression of ING1b. Error bars, SD. *p < 0.05. **d** ChIP assay performed with primers specific to downstream of promoters of *CDKN1A* and *BAX* did not show any enrichment and was used as a control to rule out false-positive in results obtained in **c** due to incomplete DNA fragmentation. Error bars, SD. **e** ChIP confirmed ING1b mediates p53-mediated transcriptional activation of *CDKN1A* and *BAX*. Significant attenuation of direct interaction with either promoter was observed in YD-9 and YD-8 cells stably transduced with ING1b shRNA. Error bars, SD. *pP < 0.05. **f** ChIP assay performed with primers specific to downstream of promoters of *CDKN1A* and *BAX* did not show any enrichment and was used as a control to rule out false-positive in results obtained in **e** due to incomplete DNA fragmentation. Error bars, SD

We next determined if the changes in acetylated p53 post-modulation of p33ING1b protein expression observed and changes in Bax and p21 (Figs. 1 and 2) were due to direct transactivation of Bax and p21 by acetylated p53. Firefly luciferase expressing promoter constructs for *CDKN1A* (encoding p21) and *BAX* were co-transfected along with renilla luciferase control plasmids in YD-9 and YD-8 cells either overexpressing p33ING1b (Fig. 3a) or shRNA targeting p33ING1b (Fig. 3b). Over-expression of p33ING1b significantly increased reporter activity for both *CDKN1A* and *BAX* (Fig. 3a; P < 0.05 in each case), whereas knockdown of p33ING1b significantly downregulated reporter activity of both *CDKN1A* and *BAX* (Fig. 3b; P < 0.05 in each case) in both YD-9 and YD-8 cells. This indicated that expression level of p33ING1b protein is correlated to transcriptional activation of both *BAX* and *CDKN1A*. We next performed ChIP assays to determine if the changes in transcriptional activation of *BAX* and *CDKN1A* was due to differences in level of acetylated p53 following modulation of expression of ING1b. Immunoprecipitation using antibody against acetylated p53 showed significant enrichment of *BAX* and *CDKN1A* in both YD-9 and YD-8 cells overexpressing p33ING1b (Fig. 3c; P < 0.05 in each case). No enrichment was observed when primers specific to downstream regions of the *BAX* and *CDKN1A* primers were used (Fig. 3d) confirming specificity of the ChIP assay. Conversely, in YD-9 or YD-8 cells in which p33ING1b expression has been knocked down, ChIP assay showed significant attenuation of direct interaction with both *BAX* and *CDKN1A* promoters (Fig. 3e; P < 0.05 in each case). Again, no enrichment was observed when primers specific to downstream regions of the *BAX* and *CDKN1A* primers were used (Fig. 3f) confirming specificity of the ChIP assay. Given that modulation of p33ING1b expression impacts acetylation of p53 (Figs. 1 and 2), these results taken together provide evidence that expression level of p33ING1b is correlated to acetylation of p53 and subsequent transactivation of *BAX* and *CDKN1A*. These results also indicate that the correlation is independent of p53 mutation status.

**Fig. 4:**
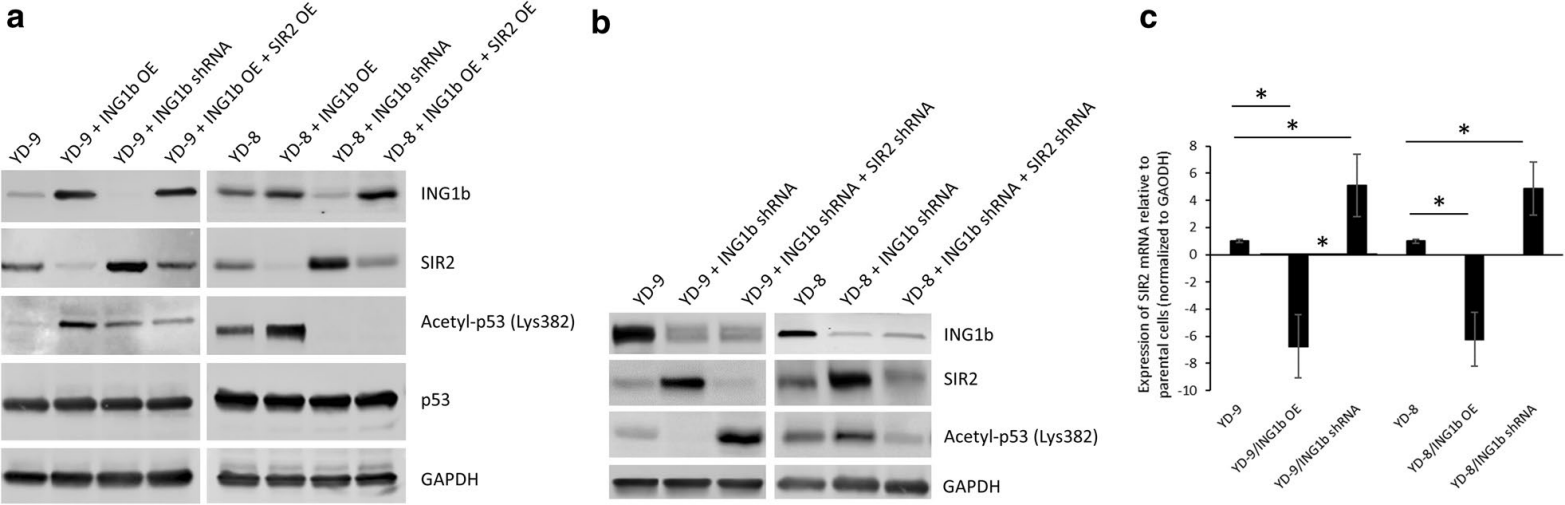
ING1b upregulates acetylated-p53 by modulating SIR2 levels. **a** Immunoblot analysis of ING1b, SIR2, acetylated and total p53 in YD-9 and YD-8 cells either transiently overexpressing ING1b alone or along with SIR2 or stably transduced with ING1b shRNA. GAPDH was used as a loading control. Data is representative of three independent experiments. **b** Effect of ING1b on p53 acetylation is mediated via SIR2. Immunoblot analysis of ING1b, SIR2, and acetylated p53 in YD-9 and YD-8 cells stably transduced with control shRNA, ING1b shRNA, or both ING1b and SIR2 shRNA. GAPDH was used as a loading control. Data is representative of three independent experiments. **c** Quantitative real-time PCR analysis of relative expression of SIR2 mRNA in YD-9 and YD-8 cells in which ING1b is overexpressed or knocked down. Data shown was normalized to GAPDH mRNA expression and expressed relative to parental YD-9 or YD-8 cells, respectively. Error bars, SD. *P < 0.05

Given that deacetylation of p53 has been shown to be a target of the NAD-dependent deacetylase silent information regulator 2 (SIR2), we next determined if modulating expression levels of p33ING1b expression is altering expression of SIR2. Overexpression of p33ING1b in YD-9 or YD-8 cells decreased expression of SIR2 and increased acetylation of p53 (Fig. 4a). Conversely, knockdown of p33ING1b increased expression of SIR2 and decreased acetylation of p53 in the YD-9 and YD-8 cells (Fig. 4a). We next overexpressed both p33ING1b and SIR2 together in the YD-9 and YD-8 cells. Acetylation of p53 in YD-9 or YD-8 cells co-overexpressing both p33ING1b and SIR2 was low, similar to that observed in YD-9 or YD-8 cells stably expressing ING1b shRNA (Fig. 4a). These results indicated that SIR2 was functioning downstream of p33ING1b. We next confirmed that decrease in p53 acetylation following knockdown of p33ING1b was indeed due to an increase in SIR2 expression. YD-9 or YD-8 cells stably expressing p33ING1b shRNA were transduced with shRNA targeting SIR2. In YD-9 and YD-8 cells in which p33ING1b was knocked down, SIR2 levels increased and acetylated p53 expression decreased compared to cells expressing control shRNA. However, when SIR2 was also knocked down along with p33ING1b, the expression of acetylated p53 expression was higher compared to both cells expressing control shRNA or both p33ING1b and SIR2 shRNA (Fig. 4b). This indicated that SIR2 was indeed deacetylating p53 and expression of p33ING1b was modulating p53 acetylation by its effect on SIR2. We performed quantitiative real-time PCR to analyze levels of SIR2 mRNA in YD-9 and YD-8 cells in which p33ING1b is overexpressed or knocked down in comparison to parental cells. Overexpression of p33ING1b significantly decreased while its knockdown significantly increased SIR2 mRNA expression in both YD-9 and YD-8 cells (Fig. 4c; P < 0.05 in each case). This indicated that p33ING1b transcriptionally regulates expression of SIR2; however, whether it is a direct regulation or involves additional cofactors remains to be determined in future studies.

**Fig. 5:**
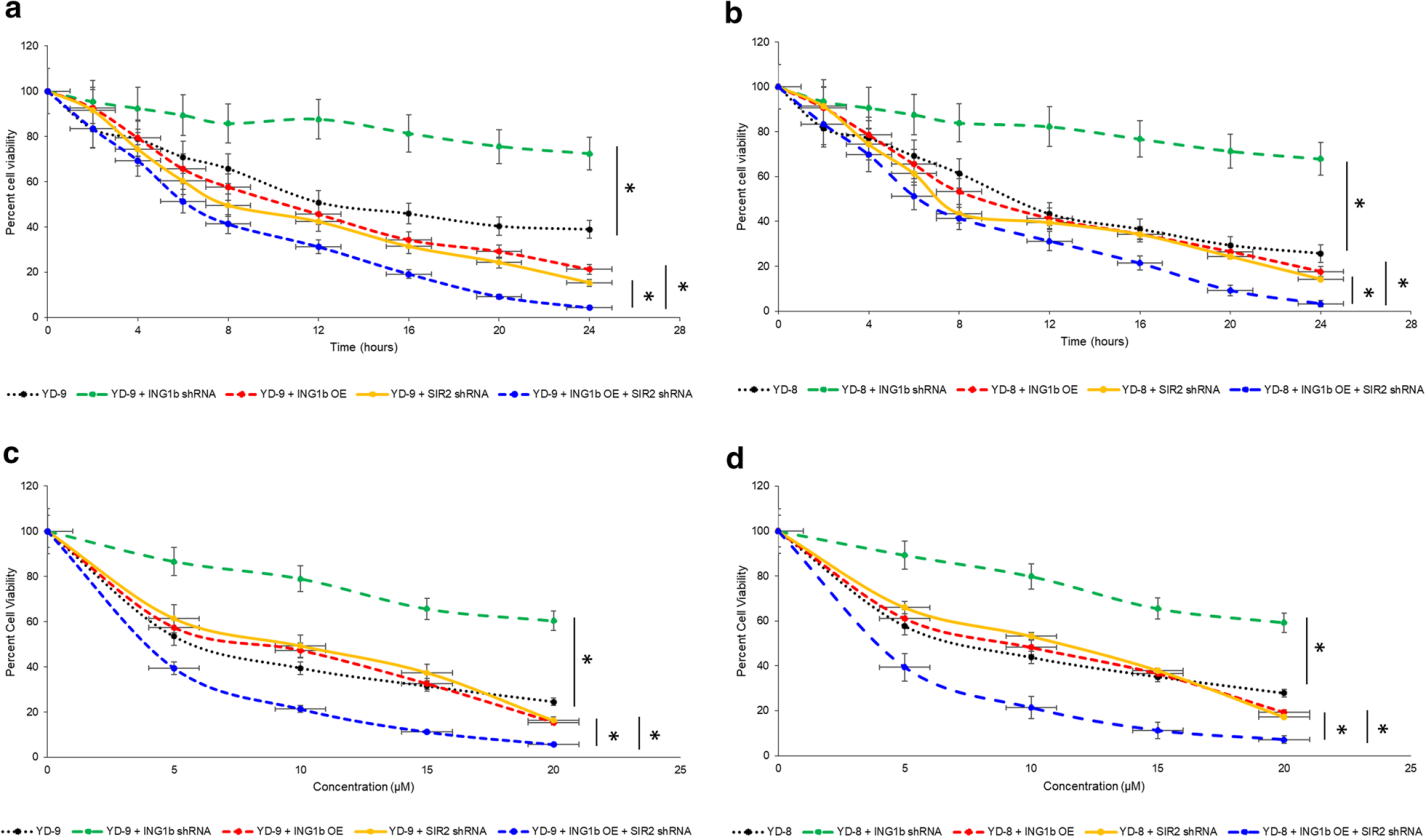
Synergistic effect of ING1b and SIR2 on chemosensitivity of YD-9 and YD-8 cells to cisplatin. Parental YD-9 **a** or YD-8 **b** cells, or cells transiently transfected with ING1b expression plasmid, stably transduced with *ING1b* shRNA, stably transduced with *SIR2* shRNA or cells overexpressing ING1b and in which SIR2 has been stably knocked down, were treated with 5 µM of cisplatin and cell viability was measured after indicated times. Same as **a** and **b**, but YD-9 cells **c** or YD-8 cells **d** were treated with indicated concentrations of cisplatin for 12 h. All data is representative of three independent experiments, each done in triplicate. Error bars, SD. *P < 0.05

We next determined if modulating expression of p33ING1b would impact chemosensitivity of the YD-9 and YD-8 cells to cisplatin. Mimicking the effect of knockdown of p33ING1b on BAX and p21 (Figs. 1, 2 and 3), knockdown of p33ING1B significantly decreased chemosensitivity to cisplatin, both when tested over a time course in YD-9 (Fig. 5a) and YD-8 (Fig. 5b) cells. Similar results were observed with increasing dosage in both YD-9 and YD-8 cells (Fig. 5c, d). Conversely, overexpression of p33ING1b significantly increased chemosensitivity to cisplatin (Fig. 5a–d). Similarly, knockdown of SIR2 significantly increased chemosensitivity to cisplatin in both YD-9 and YD-8 cells (Fig. 5a–d). Importantly, concomitant overexpression of p33ING1b and downregulation of SIR2 had a significant synergistic effect on chemosensitivity to cisplatin in both YD-9 and YD-8 cells, compared to either p33ING1b overexpression or SIR2 shRNA (Fig. 5a–d; P < 0.05 in each case). Taken together this indicated that modulating expression of either p33ING1b or SIR2 alone or in combination can have potential therapeutic benefit in increasing chemosensitivity of OSCC cells to cisplatin treatment.

## Discussion

Genomic alteration in chromosome 12q33-34, where ING1 is located, or decrease in expression of p33ING1b in OSCC patients have been reported [25, 26, 29]. These incidences vary between 7 and 68% of cases [25, 26, 29], the wide variability can be due to study parameters and number of patients included in these studies. Taken together with the high prevalence of p53 mutation in OSCC patients, it might be possible that differences in nuclear and cytoplasmic shuttling of p33ING1b along with its effect on acetylation of p53 as observed in this study might be how differential p33ING1b expression still plays a pathogenic role in OSCC. One limitation of the current study is with the experiments performed we cannot rule out completely the possibility of any altered function of ING1b in YD-8 cells. To assert that comprehensive analysis of genomic organization of ING1b in YD-8 and other OSCC cell lines along with detailed functional characterization needs to be performed.

R273 is one of the mutation hotspots in p53, with p.R273H, p.R273C, and p.R273G variants normally observed in different tumors [30]. The p.R273H variant has been shown to enhance cancer cell malignancy [30]. The p.R248W and p.R248Q mutations in Ca9-22 and Sa-3 cell lines, respectively, both result in gain-of-function of p53 like the p.R273H mutation [31]. Normally acetylation of p53 is connected to transactivation of its pro-apoptotic downstream targets. Given that (a) our results show that p33ING1b expression is correlated to acetylation of p53 in the tested OSCC cell lines irrespective of their mutation status, (b) gain-of-function p53 mutation favors cancer progression unlike wild type p53, and (c) our observation that OSCC cell lines with mutated p53 have relatively high basal levels of acetylated p53 expression, it will be important to investigate how OSCC cell lines with mutant p53 circumvent the pro-apoptotic functions of increased acetylated p53.

SIR2 is a class III histone deacetylase and along with other sirtuins function in cell proliferation, aging and cell metabolism [32–34]. SIR2 has been indicated in chemoresistance, so it was not surprising to find that knockdown of SIR2 increased chemosensitivity of the YD-9 cells. However, our results show that downregulation of SIR2 is increasing chemosensitivity in part by increasing acetylation and transactivation of p53 signaling and that basally SIR2 expression is regulated by p33ING1b.

However, the exact role of SIR2 in OSCC and tumorigenesis in general is not well known. It has been though found to be overexpressed in multiple cancer types [35–37] and can potentially function by suppressing p53 function as our results show or driving the function of other tumor drivers [38]. The role of SIR2 in OSCC is largely unknown, except for one study in which it was suggested as a tumor suppressor [39]. Our results corroborate this finding. Our results show that it is expressed in OSCC cell lines and that its downregulation might be therapeutically beneficial.

## Conclusions

Our results indicate that expression of SIR2 and p33ING1b are connected to cell proliferation and chemosensitivity in the context of OSCC cell lines. Given that modulation of p33ING1b increased chemosensitivity to cisplatin in YD-9 cells, it remains to be determined if overexpression in other OSCC cell lines will have a similar effect. More importantly, using in vivo models of OSCC it needs to be determined if adenovirus-mediated overexpression of p33ING1b will have a favorable outcome on disease progression. Furthermore. it needs to be determined if knockdown of SIR2 in this context will have a synergetic therapeutic benefit as we observed in our in vitro studies.

## Data Availability

All data generated or analyzed during this study are included in this published article.

## Abbreviations

OSCC: Oral squamous cell carcinoma
HNSCC: Head and neck squamous cell carcinoma
PHD: The plant homeodomain
SIR2: Silent information regulator 2
PCNA: Proliferating nuclear cell antigen
